# Bovine Milk Extracellular Vesicles as a Preventive Treatment for Bone Dysfunction and Metabolic Alterations in Obese Mice Fed a High‐Refined Carbohydrate Diet

**DOI:** 10.1002/mnfr.70139

**Published:** 2025-07-02

**Authors:** Francine R. F. Silva, Joyce E. Heredia, Onno J. Arntz, Breno R. Barrioni, Mauro M. Teixeira, Tarcília A. Silva, Fons A. J. van de Loo, Soraia Macari, Adaliene V. M. Ferreira, Marina C. Oliveira

**Affiliations:** ^1^ Immunometabolism, Department of Nutrition Nursing School Universidade Federal de Minas Gerais Belo Horizonte Minas Gerais Brazil; ^2^ Experimental Rheumatology Radboud University Medical Center Nijmegen the Netherlands; ^3^ Department of Chemical and Materials Engineering School of Engineering Universidade Federal de Lavras Lavras Minas Gerais Brazil; ^4^ Immunopharmacology Department of Biochemistry and Immunology Institute of Biological Sciences Universidade Federal de Minas Gerais Belo Horizonte Minas Gerais Brazil; ^5^ Department of Oral Surgery and Pathology Faculty of Dentistry Universidade Federal de Minas Gerais Belo Horizonte Minas Gerais Brazil; ^6^ Department of Restorative Dentistry Faculty of Dentistry Universidade Federal de Minas Gerais Belo Horizonte Minas Gerais Brazil

**Keywords:** bone loss, extracellular vesicles, metabolism, milk, obesity

## Abstract

Obesity may cause bone loss due to changes in energy and bone metabolism. Bone loss treatment is still limited, requiring new therapeutic strategies. Bovine milk extracellular vesicles (MEVs) are nanoparticles that act as modulators of cell signaling. While its benefits have already been demonstrated in bone loss, the underlying mechanisms must be better elucidated. To evaluate the effect of MEVs on bone loss in obesity, BALB/c mice were fed a chow diet or a high‐refined carbohydrate (HC) diet for 12 weeks and treated or not with MEVs from the 9th week. Mice fed the HC diet showed bone loss in the maxillary and long bones related to detrimental changes in the bone cell profile. As expected, the HC diet induced hyperglycemia and dyslipidemia, lipid accumulation in adipose and liver, and elevated receptor activator of nuclear factor kappa‐Β ligand (RANKL)/osteoprotegerin (OPG) ratio and Pentraxin 3 (PTX3) levels. MEV treatment protected from bone loss, increasing osteoblast and osteocyte numbers and reducing osteoclastic activity. Additionally, MEVs diminished adiposity, liver damage, serum glucose, triglyceride, and PTX3 levels, and the RANKL/OPG ratio. MEVs offer a protective effect against bone loss and improve metabolic outcomes in an HC diet‐induced obesity model, suggesting that metabolic improvements may contribute to their bone‐protective role.

## Graphical abstract

Obesity induction through a high‐refined carbohydrate (HC) diet in BALB/c mice led to increased bone resorption and bone loss in the femur and maxilla. This diet also resulted in increased adiposity, glucose and lipid levels, and insulin resistance. Treatment with bovine milk extracellular vesicles (MEVs) in HC diet‐fed mice demonstrated reduced bone loss and bone resorption associated with decreased adiposity, glucose and lipid levels, and insulin resistance. Therefore, these results demonstrate the promising potential of MEVs as a protective approach to simultaneously address the skeletal and metabolic dysfunction induced by the HC diet. *Created in BioRender. Ferreira, A. (2025)* https://BioRender.com/n11y635.

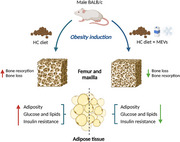

**Abbreviations**
CEJ‐ABC: cemento‐enamel junction and alveolar bone crest
BMD: bone mineral density
BV/TV: bone volume per total tissue volume
EAT: epididymal adipose tissue
HC: high‐refined carbohydrate
MEV: bovine milk extracellular vesicle
RMD: root mineral density
RV/TV: root volume per total tissue volume
SAT: subcutaneous adipose tissue
SMI: structure model index
Tb.N: number of trabeculae
Tb.Sp: trabecular separation
Tb.Th: trabecular thickness

## 1 Introduction

Obesity is a multifactorial chronic disease characterized by excessive fat accumulation and related to the development of distinct metabolic changes, such as insulin insensitivity, hyperglycemia, ectopic fat deposition, dyslipidemia, and bone loss [1, 2]. Excessive consumption of nutrients, such as refined carbohydrates and saturated fatty acids, leads to hypertrophy of fat cells, which, through the production of inflammatory mediators, alters not only energy metabolism but also the functioning of bone cells [2, 3, 4, 5, 6]. This imbalance in bone remodeling, favoring resorption over formation, contributes to structural bone loss. These changes may lead to osteoporosis, a disease in which bone suffers a marked bone renewal, with more bone resorption to the detriment of bone formation, resulting in brittle bone and more likely to fracture [7, 8, 9, 10].

Current bone loss adjuvant treatments are limited and not consistently effective, such as medications associated with calcium and vitamin D supplementation [9, 11]. Diet is essential in the prevention and treatment of obesity and its bone‐related complications by an adequate intake of calories, proteins, calcium, and vitamin D [12, 13]. However, diet therapy is still insufficient as an adjuvant treatment for bone loss [14, 15]. Therefore, these limitations underscore the need for emerging alternative or complementary strategies to mitigate both metabolic and skeletal dysfunctions in obesity.

In this context, extracellular vesicles (EVs), particularly derived from bovine milk, have emerged as a promising area of investigation. Bovine milk extracellular vesicles (MEVs) are nanoparticles derived from the plasma membrane of mammary gland cells that, after being secreted, can act as potent cellular signaling due to their content of proteins, lipids, coding and noncoding RNA [16, 17, 18]. These structures have demonstrated positive effects on the functioning of bone cells in experimental models of bone dysfunction, such as ovariectomy, use of glucocorticoids, and diet, protecting against bone loss [19, 20, 21]. Also, MEVs were demonstrated to increase osteogenesis, regulate osteoclastic activity, and influence bone remodeling markers, resulting in positive effects for bone protection [22, 23, 24]. However, their mechanisms have not yet been fully elucidated. Despite these promising results, it is still necessary to understand the impact of MEVs in the context of obesity‐induced alterations in bone and systemic metabolism.

Given the immunometabolic dysfunction involved in the pathology of obesity that compromises bone health and remodeling, it is desired to have a full treatment that may act in a wider perspective, aiming at better outcomes in both fronts. Then, new therapeutic strategies must be investigated. Considering the potential of MEVs to modulate the activity of bone cells, we hypothesized that these nanoparticles could also improve metabolic and bone‐related parameters in a model of obesity‐induced bone loss. Thus, this study aimed to evaluate the effects of oral administration of MEVs as a therapeutic strategy in mice treated with a diet rich in refined carbohydrates (high‐refined carbohydrate, HC), focusing on the impact of these vesicles on both metabolic and bone parameters.

## 2 Materials and Methods

### 2.1 Experimental Protocol and Treatments

Eight‐week‐old male BALB/c mice (*n* = 18) were maintained in a controlled environment with a 12‐hour light/dark cycle, with ad libitum access to both water and food throughout the entire 12‐week period, being randomly divided into three groups (*n* = 6 for each group): (i) Control Group (C) fed standard chow diet (Labina); (ii) HC group fed a diet rich in refined carbohydrates; (iii) Treated group (HC‐MEVs) fed a diet rich in refined carbohydrates and treated with bovine MEVs. The HC diet was prepared by modifying the commercial Labina diet, adding condensed milk and refined sugar, with around 30% of the calories from sucrose [25]. The nutritional information for diets is presented in Table S1.

The animals were fed the respective diets throughout the experimental period, without food or water restrictions, and weighed weekly to assess weight gain. After 12 weeks, the mice were euthanized by exsanguination following anesthesia with ketamine (80 mg/kg) and xylazine (10 mg/kg) diluted in 0.9% NaCl. Blood was collected to assess systemic energy metabolism, bone turnover, and inflammation in the serum. Visceral and subcutaneous adipose tissue and the liver are key metabolic organs affected by obesity. Then, we collected and weighed epididymal (visceral) and inguinal (subcutaneous) adipose tissues and the liver. The femur and maxilla represent long and short bones, respectively, allowing evaluation of bone quality under different mechanical load conditions. All experimental procedures were approved by the Ethics Committee on Animal Use of the Federal University of Minas Gerais (CEUA/UFMG), under protocol number 182/2023.

### 2.2 Dosage Information and Extraction of MEVs

MEVs were offered to mice orally starting in the 9th week of diet‐induced obesity, adding a solution to drinking water with 14.3 × 10^6^/mL of particles (approximately 143 nanograms of protein/mL) throughout the treatment until week 12, lasting for 4 weeks. The selected dosage was based on previously published preclinical studies that demonstrated beneficial effects of orally administered bovine milk‐derived extracellular vesicles on arthritis and bone without adverse effects [19, 21, 22, 23, 26]. Based on an estimated water intake of 5 mL/day for a mouse and average body weight of 25 g, the daily dose corresponded to 28.6 µg of EV protein/kg of body weight (human equivalent dose [HED] of 162 µg protein/day for a 70‐kg person). Drinking water was replaced every 2 to 3 days. The control and HC group received the MEVs vehicle (phosphate‐buffered saline—PBS) at an equivalent volume dose in the same period.

MEVs were obtained from the Experimental Rheumatology Laboratory (Radboudumc, Nijmegen, the Netherlands), where particle isolation was carried out. For this, a sample of semiskimmed commercial milk was used and subjected to a successive ultracentrifugation process, as described by Pieters et al. [18]. Briefly, the milk was centrifuged at 70 000 × *g* for 1 h at 4°C to remove fat globules, proteins, and other substances. The transparent part of the supernatant was filtered through Whatman paper No. 1, followed by No. 50. Then, the effluent was filtered with a syringe filter (0.22 µm). This effluent was centrifuged at 100 000 × *g* for 1.5 h at 4°C. The pellet was washed 4 times with PBS on top, and each wash step was collected. The total volume was filtered again using a 0.22 µm syringe filter. The amount of protein was measured with a Micro‐BCA kit (Thermo Scientific, Pierce, Rockford, IL, USA), and proteomic analysis was shown previously, along with the exosome marker CD63, miRNA, and milk‐specific proteins [26]. Additional characterization was performed using nanoparticle tracking analysis (NS300), and the MEVs had an average diameter of approximately 140 nm. Images of bovine MEVs by cryo‐electron microscopy (cryo‐EM) are shown in Figure S1. The MEVs protein per particle concentration was 10 fg. According to the International Society of Extracellular Vesicles (ISEV), MEVs have all the characteristics of exosomes [27].

### 2.3 Microcomputed Tomography (micro‐CT) Analysis

For bone analysis, the femur and jaws of the animals were scanned using a high‐resolution microcomputed tomography (micro‐CT) scanner (x‐ray microtomography Skyscan 1174, Aartselaar, Belgium). The images obtained by scanning were reconstructed with the NRecon software (Skyscan, Belgium). The bones were positioned to standardize the area analyzed using the DataViewer software (Skyscan, Belgium) and subsequently analyzed using the CTAn software (Skyscan, Belgium). The parameters of bone mineral density (BMD), bone volume per total tissue volume (BV/TV), trabecular thickness (Tb.Th), number of trabeculae (Tb.N), trabecular separation (Tb.Sp), model structure (SMI), total area within the periosteum (Tt.Ar), bone cortical area (Ct.Ar), cortical area fraction (Ct.Ar/Tt.Ar) and cortical thickness (Ct.Th) were performed in a maxillary and femoral region of interest (ROI). Root evaluation was performed as previously described [28]. Root mineral density (RMD) and root volume per total tissue volume (RV/TV) were analyzed in all root extensions in the mesiobuccal, distobuccal, and palatal roots of first molars with irregular anatomical regions of interest drawn manually. The height of alveolar bone crest loss was determined by measuring the distance between the alveolar bone crest (ABC) and the cementoenamel junction (CEJ), known as CEJ‐ABC in three‐dimensional images, from the mesial surface of the first molar to the distal surface of the maxillary third molar on the palatal surface using ImageJ software (National Institutes of Health, MD, USA).

### 2.4 Histomorphometric Analysis

All tissues were dissected and fixed with a 4% buffered formalin solution for histological processing. Adipose and liver tissues were dehydrated, cleared, and embedded in paraffin. Histological sections of 5 µm thickness stained with hematoxylin–eosin (H&E) were obtained to analyze the adipocyte area and the histopathological score of the liver using ImageJ software (NIH Image, Bethesda, MD, USA). The maxilla and femur underwent the decalcification process with EDTA and were impregnated with paraffin. H&E staining was used to assess the number of osteocytes, Masson's Trichrome staining was used to evaluate the presence of osteoblasts, and a TRAP staining kit assessed osteoclasts. The analyses were carried out using a specific camera (Carl Zeiss, Göttingen, Niedersachsen, Germany) with an attached digital camera (PowerShot A620, Canon, Tokyo, Honshu, Japan). ImageJ (NIH Image, Bethesda, MD, USA) software was used for the analyses.

To evaluate the size of adipocytes, the cellular area of all adipocytes with well‐defined boundaries was measured in five histological sections per animal under 100× magnification. The liver histopathological score was performed by evaluating hepatic steatosis, inflammatory infiltrate, and ballooning of hepatocytes under 400× magnification. Each feature was scored individually based on the degree of alteration: (1) absent, (2) minimal presence, (3) moderate presence, (4) marked presence. The final score was obtained by summing the values for all three parameters, providing an overall measure of liver histopathological alterations.

For the analysis of bone tissue, the number of osteoblasts was determined along the length of the alveolar bone and femur bone trabeculae in a given area. Osteocytes were quantified by considering the number of lacunae in an alveolar bone or trabeculae of the femur and measuring the total bone area in the analysis region. Osteocyte density was calculated by dividing the number of cells/gaps counted by the area of the alveolar bone or femur. Osteoclast counts were performed along the length of the alveolar bone in the furcation region of the first molar and the trabeculae area of the distal metaphysis of the femur.

### 2.5 Serum Analyses

The markers for bone remodeling, receptor activator of nuclear factor kappa‐Β ligand (RANKL), related to osteoclast differentiation, and osteoprotegerin (OPG), a blocker of RANKL, also the adipokines mainly produced by adipose tissue, adiponectin and leptin, and Pentraxin 3 (PTX3), a maker for systemic inflammation were quantified in the serum of mice using DuoSet ELISA kits (R&D Systems Europe Ltd., Abington, UK). A serum insulin ELISA kit was provided by Millipore (St. Louis, MO, USA). Serum levels of glucose, total cholesterol, triglycerides, alanine aminotransferase (ALT), aspartate aminotransferase (AST), and gamma‐glutamyl transferase (GGT) were assessed using enzymatic kits (Bioclin, Belo Horizonte, MG, Brazil). Analyses were performed according to the manufacturer's instructions.

### 2.6 Real‐Time Polymerase Chain Reaction (PCR)

According to the manufacturer's instructions, total RNA was isolated from bone and adipose tissue using the Invitrogen PureLinkTM RNA Mini Kit (Life Technologies Corp, North America). A lysis buffer containing 2‐mercaptoethanol was added to the samples, followed by homogenization with a rotor‐stator homogenizer (IKA, Staufen, Germany). Ethanol was added to the lysate, and the solution was transferred to the extraction column. After centrifugation, the RNA was successively washed with wash buffer. Finally, the RNA was eluted with RNAse‐free water. Transcription of the RNA was performed to obtain cDNA. For this purpose, a thermocycler (BioRad T100, Hercules, California, USA) and the iScript cDNA Synthesis reverse transcriptase enzyme (BioRad T100, Hercules, California, USA) were used. The ViiATM 7 Real‐Time PCR System (Thermo Fisher Scientific, MA, USA) was used for the analysis, and the SYBR Green PCR Master Mix kit (Thermo Fisher Scientific) was used. The gene expression of the following markers was evaluated: RUNX2, related to osteoblastogenesis, and PPARγ, related to adipogenesis. These two markers are functionally interconnected in bone, as PPARγ can inhibit osteoblastogenesis by suppressing RUNX2 activity, while RUNX2 can limit adipogenic differentiation by downregulating PPARγ. Also, PPARγ is essential in adipose tissue for adipocyte differentiation, lipid storage, insulin sensitivity, and inflammation control. Gene expression levels were determined by the 2−ΔΔCt method and were normalized to the expression of the glyceraldehyde‐3‐phosphate dehydrogenase gene (GAPDH). The sequences of primer pairs are listed in Table S2.

### 2.7 Statistical Analysis

Mice were randomly divided into groups in cages of six animals. We tested that the mean body weight was statistically not different for each experimental group before assignment to diet treatment. The normality test verified that the samples have a Gaussian distribution. Statistical comparisons were done using “one‐way” ANOVA followed by Dunnett posttest. The sample size was calculated with GPower Software (version 3.1.9.7). To calculate the sample size, we used ANOVA one‐way with five experimental groups, effect size = 0.8, *α* error = 0.05, and statistical power = 0.7. The total sample size was 18 animals = 6 mice in each group. All tests and histology analyses were performed blinded by investigators. Each experimental group initially comprised 6animals (*n* = 6). Due to occasional sample loss during processing, some analyses were conducted with 4 to 6 animals per group (*n* = 4–6), as specified in the corresponding figure legends. The Grubbs' test was performed to determine outliers among the samples, and values statistically lower than 0.05 were considered atypical and excluded from analyses. The results are presented as mean ± standard error of the mean, with a significance level of *p* < 0.05. Analyses were performed using the GraphPad PRISM 8.0 software (GraphPad Software Inc., San Diego, CA, USA).

## 3 Results

### 3.1 MEVs Protected Alveolar Bone From HC Diet‐Induced Bone Loss by Modulating the Recruitment/Differentiation of Bone Cells in the Maxilla

**FIGURE 1:**
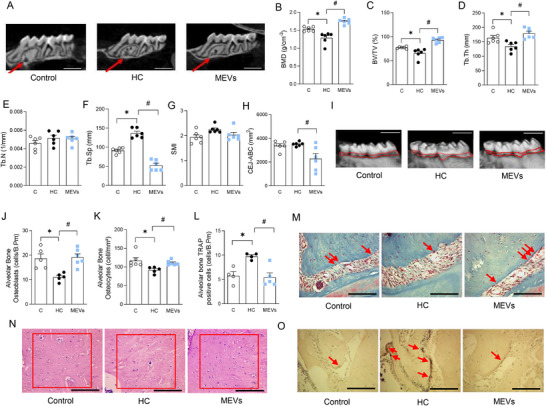
Analysis of the maxillary alveolar bone of mice by computed microtomography and histomorphometry. (A) Representative maxillary image of computed microtomography analysis, (B) BMD, (C) BV/TV, (D) Tb.Th, (E) Tb.N, (F) Tb.Sp, (G) SMI, (H) CEJ‐ABC, (I) Representative CEJ‐ABC image. (*n* = 6 per group). Micro‐CT scale bars, 25 µm. (J) Osteoblast, (K) Osteocyte and (L) TRAP positive cell counts in alveolar bone by histomorphometry. Representative image of (M) osteoblasts, (N) osteocytes, and (O) TRAP cells in the alveolar bone (400×, histological scale bars, 100 µm) of mice fed a control diet or a diet rich in refined carbohydrates (HC) for 12 weeks and treated with bovine MEVs in the last 4 weeks (*n* = 4–6 per group). Bars represent mean values ± standard error of the mean. Statistical difference represented by *p < 0.05–HC vs. Control and #*p* < 0.05–HC vs. MEVs, one‐way ANOVA, Dunnett posttest for all data. BMD, bone mineral density; BV/TV, percent bone volume/tissue volume; CEJ‐ABC, cementum–enamel junction to alveolar bone crest; MEV, milk extracellular vesicle; SMI, structure model index; Tb.N, number of trabeculae; Tb.Sp, separation between trabeculae; Tb.Th, trabecular thickness.

The water intake did not change among the experimental groups, independent of the treatments (C: 4.66 ± 0.20; HC: 4.30 ± 0.24; MEVs: 4.49 ± 0.12 mL/day/mouse; *p* > 0.05). Then, each mouse treated with MEVs consumed around 6.4 × 10^7^ of particles per day. We initially evaluated the maxilla due to its high remodeling rate and direct exposure to dietary changes. Microcomputed tomography analysis of the maxilla revealed that animals fed the HC diet had lower BMD, BV/TV, and Tb.Th, along with higher Tb.Sp compared with the control group (Figure 1A–D,F). Notably, MEV treatment mitigated these effects, as mice in the MEV group showed improvements in all parameters relative to the untreated HC group (Figure 1A–D,F). Tb.N and SMI were not altered by diet or MEV treatment (Figure 1E,G). Complementarily, the CEJ‐ABC analysis showed significant differences only between the HC diet and the treated group, with the area of bone loss reduced in the last one (Figure 1H,I). The structure of the tooth roots did not show significant differences between the groups (Figure S2A–H).

The histomorphometry evaluation of the alveolar bone demonstrated that mice fed the HC diet had fewer osteoblasts and osteocytes, and a more significant presence of TRAP‐positive cells, i.e., osteoclasts, than the control group. The MEVs‐treated group showed a more substantial presence of osteoblasts and osteocytes in the maxilla, associated with lower osteoclast numbers than the HC‐untreated group (Figure 1J–O).

### 3.2 MEVs Protected Long Bone Disruption Induced by the HC Diet and Positively Influenced the Expression of RUNX2 and Systemic Bone Remodeling Markers

**FIGURE 2:**
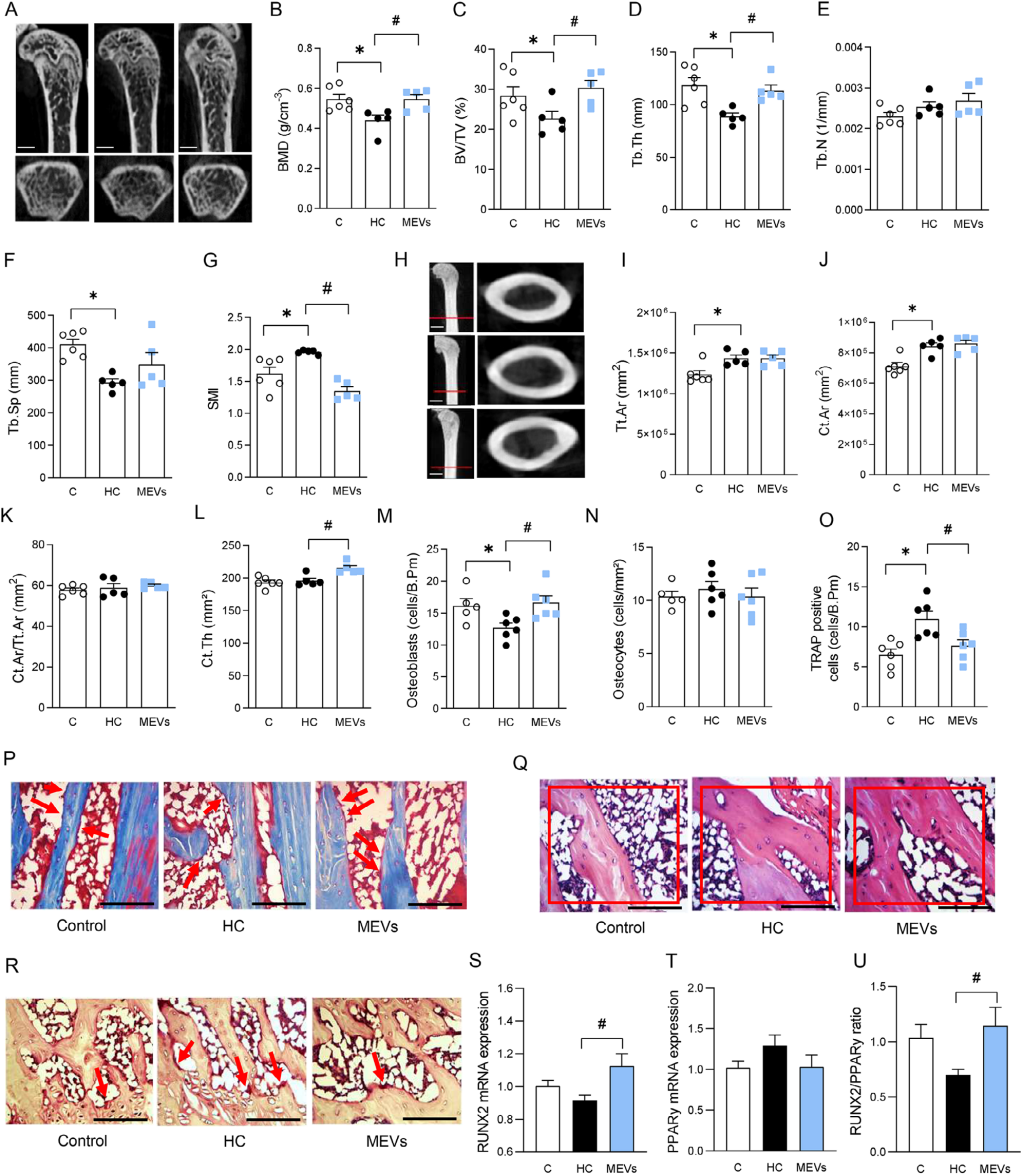
Analysis of the femur of mice by computed microtomography, histomorphometry and RT‐PCR. (A) Representative femur image of computed microtomography analysis, (B) BMD, (C) BV/TV, (D) Tb.Th, (E) Tb.N, (F) Tb.Sp, (G) SMI, (H) representative cortical femur image of computed microtomography analysis, (I) Tt.Ar. Micro‐CT scale bars, 10 µm. (J) Ct. Ar., (K) Ct.Ar/Tt.Ar, (L) average Ct.Th. (*n* = 5–6 per group). Micro‐CT scale bars, 5 µm. (M) Osteoblast count in trabecular bone by histomorphometry, (N) osteocyte count in trabecular bone by histomorphometry, (O) count positive TRAP cells in trabecular bone by histomorphometry, (P) representative image of osteoblasts in the trabecular bone (400×), (Q) representative image of osteocytes in the trabecular bone (400×), (R) representative image of TRAP cells in the trabecular bone (400×). (*n* = 5–6 per group). Histological scale bars, 100 µm. (S) RUNX2 expression in the femur by RT‐PCR, (T) PPARy expression in the femur by RT‐PCR, (U) RUNX2/PPARy ratio expression in the femur of mice fed a control diet or a diet rich in refined carbohydrates (HC) for 12 weeks and treated with bovine MEVs in the last 4 weeks (*n* = 6 per group). Bars represent mean values ± standard error of the mean. Statistical difference represented by *p < 0.05–HC vs. Control and #*p* < 0.05–HC vs. MEVs, one‐way ANOVA, Dunnett posttest for all data. BMD, bone mineral density; BV/TV, percent bone volume/tissue volume; Ct. Ar., cortical bone area; Ct.Ar/Tt.Ar, cortical area fraction; Ct.Th, cortical thickness; MEVs, milk extracellular vesicles; SMI, structure model index; Tb.N, number of trabeculae; Tb.Sp, separation between trabeculae; Tb.Th, trabecular thickness; Tt.Ar, total cross‐sectional area inside the periosteal envelope.

To better understand the effects of the HC diet on bone parameters, we extended our analysis to the femur, a weight‐bearing bone that reflects systemic alterations in bone metabolism. The femur of mice fed an HC diet presented with lower BMD, BV/TV, and Tb.Th, and Tb.Sp, and increased SMI compared to the control group (Figure 2A–D,F,G). In contrast, mice treated with MEVs showed higher BMD, BV/TV, Tb.Th, Tb.Sp, and SMI compared with HC‐untreated group (Figure 2A–D,G). Tb.N did not show any changes among the groups (Figure 2E). Regarding cortical parameters, mice fed the HC diet demonstrated reduced Tt.Ar and Ct.Ar compared to controls without alterations by MEV treatment (Figure 2H–J). No statistical differences were found in the Ct.Ar/Tt.Ar analysis among groups (Figure 2K). However, mice treated with MEVs showed increased Ct.Th compared with the HC group (Figure 2L).

The femur of mice fed the HC diet showed reduced osteoblast and increased osteoclast number compared to the control. Mice with obesity treated with MEVs had a more significant presence of osteoblasts and fewer osteoclasts in the tissue than those untreated. There were no changes in osteocytes among the groups (Figure 2M–R). The analysis of gene expression of bone markers in the femur did not demonstrate changes in the Runx2, Ppary, and Runx2/Ppary ratio values in mice fed HC diet. However, a significant increase in *Runx2* in mice treated with MEVs was observed (Figure 2S–U).

**FIGURE 3:**
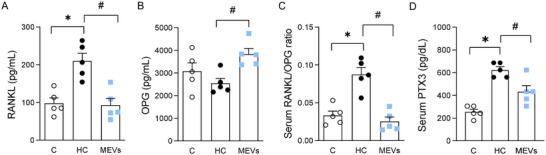
Systemic bone remodeling and inflammatory markers of mice by ELISA. (A) RANKL, (B) OPG, (C) RANKL/OPG ratio, (D) PTX3 in serum of mice fed a control diet or a diet rich in refined carbohydrates (HC) for 12 weeks and treated with bovine MEVs in the last 4 weeks (*n* = 5 per group). Bars represent mean values ± standard error of the mean. Statistical difference represented by *p < 0.05–HC vs. control and #*p* < 0.05–HC vs. MEVs, one‐way ANOVA, Dunnett posttest for all data. MEV, milk extracellular vesicle.

Systemic bone remodeling markers related to the balance between bone formation and resorption were also altered. RANKL was elevated in mice fed the HC diet, concomitant with a higher RANKL/OPG ratio than the control group (Figure 3A,C). The MEVs treatment reduced RANKL and increased OPG, consequently lowering the RANKL/OPG ratio compared with the HC group (Figure 3A–C). The OPG concentration was not different between the groups (Figure 3B). PTX3, a biomarker of systemic inflammation, was increased in mice fed HC diet compared to control, being lower in mice treated with MEVs (Figure 3D).

### 3.3 MEVs Altered Adipose Tissue Morphology and Improved Systemic Metabolism

**FIGURE 4:**
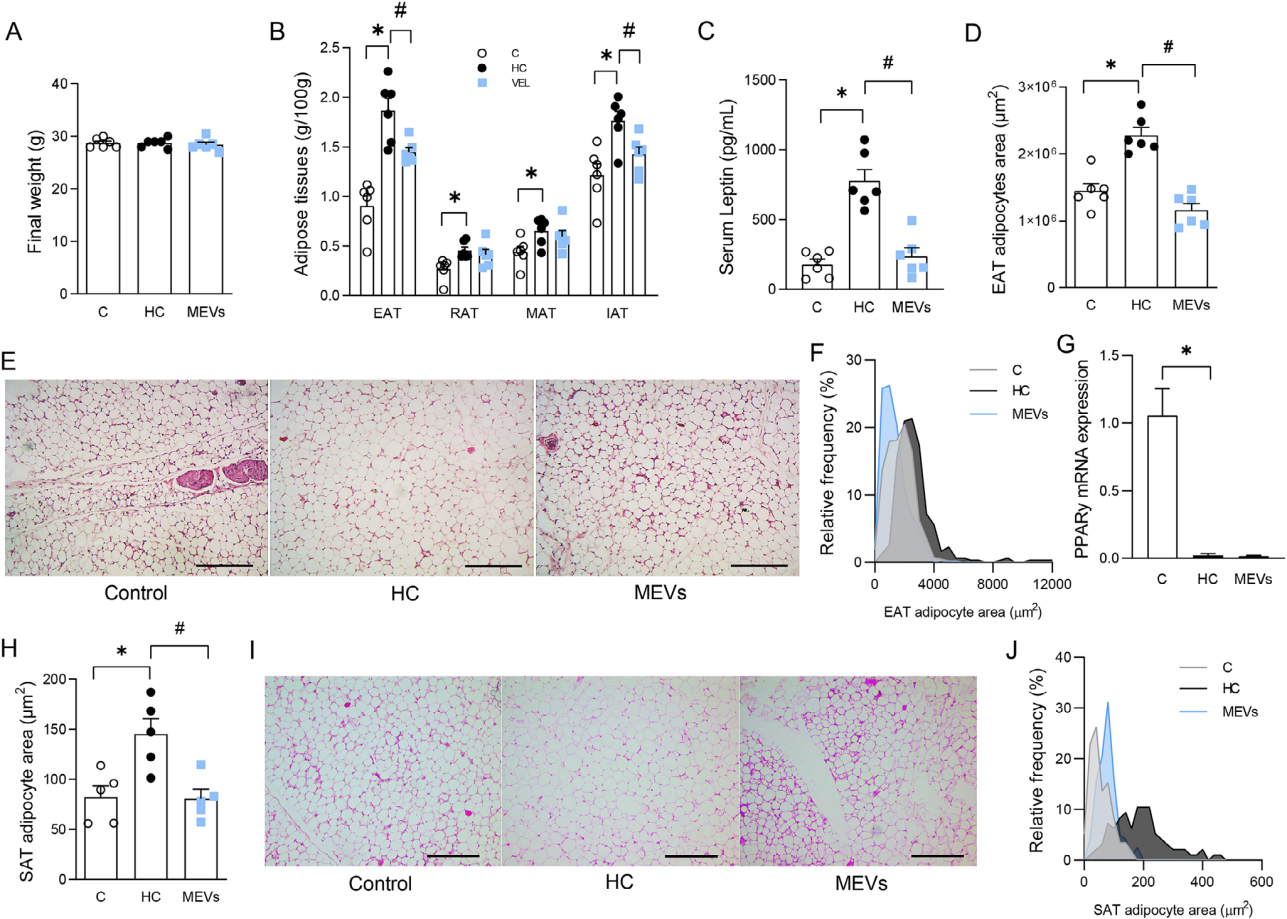
Final body weight, histological and metabolic adipose tissue analysis of mice. (A) Final weight, (B) Weight of adipose tissues: EAT, RAT, MAT, and IAT per 100 g mouse body weight, (C) Serum leptin by ELISA. (*n* = 6 per group). (D) EAT adipocyte area by histomorphometry analysis, (E) representative image of EAT adipocyte area (100×), histological scale bars, 100 µm, (F) stratification of the EAT adipocyte area, (G) EAT PPARy expression by RT‐PCR, (H) SAT adipocyte area by histomorphometry analysis, (I) representative image of the SAT adipocyte area (100×), histological scale bars, 100 µm, (J) stratification of the SAT adipocyte area of mice fed a control diet or a diet rich in refined carbohydrates (HC) for 12 weeks and treated with bovine MEVs in the last 4 weeks (*n* = 5 per group). Bars represent mean values ± standard error of the mean. Statistical difference represented by *p < 0.05–HC vs. control and #*p* < 0.05 ‐ HC vs. MEVs, one‐way ANOVA, Dunnett posttest for all data. EAT, epididymal; IAT, inguinal; MAT, mesenteric; MEV, milk extracellular vesicle; RAT, retroperitoneal.

To further investigate whether MEVs have some effect on overall metabolism, we analyzed the adipose tissue morphometry, lipid storage, hepatic alterations, and systemic metabolic parameters. The final weight of the mice showed no significant differences between groups (Figure 4A). However, the mass of epididymal, retroperitoneal, mesenteric, and inguinal adipose tissue was increased in the HC diet‐fed mice compared with the control group (Figure 4B). According to that, leptin levels were increased in this group (Figure 4C). Akin, the area of adipocytes in EAT and SAT was larger, and the bigger adipocytes more frequently spread in the adipose tissue (Figure 4D–F,H–J). Mice treated with MEVs showed diminished adipose tissue mass only in EAT and IAT (Figure 4B). This reduction was associated with lower levels of leptin (Figure 4C) and smaller medium area of EAT and SAT adipocytes, as well as a more significant number of smaller adipocytes according to the analysis of the stratification by the size of these cells compared to the HC group (Figure 4D–F,H–J). The *Ppary* gene expression in EAT demonstrated a reduction in mice fed the HC diet that was not altered by the treatment with MEVs (Figure 4G).

**FIGURE 5:**
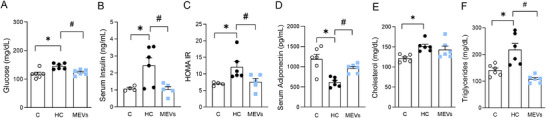
Systemic analysis in serum of mice by enzymatic tests and ELISA. (A) Glucose, (B) insulin by ELISA, (C) HOMA IR, (D) adiponectin, (E) total cholesterol, and (F) triglycerides in the serum of mice fed a control diet or a diet rich in refined carbohydrates (HC) for 12 weeks and treated with bovine MEVs in the last 4 weeks (*n* = 4–6 per group). Bars represent mean values ± standard error of the mean. Statistical difference represented by *p < 0.05–HC vs. control and #*p* < 0.05–HC vs. MEVs, one‐way ANOVA, Dunnett posttest for all data. MEV, milk extracellular vesicle.

**FIGURE 6:**
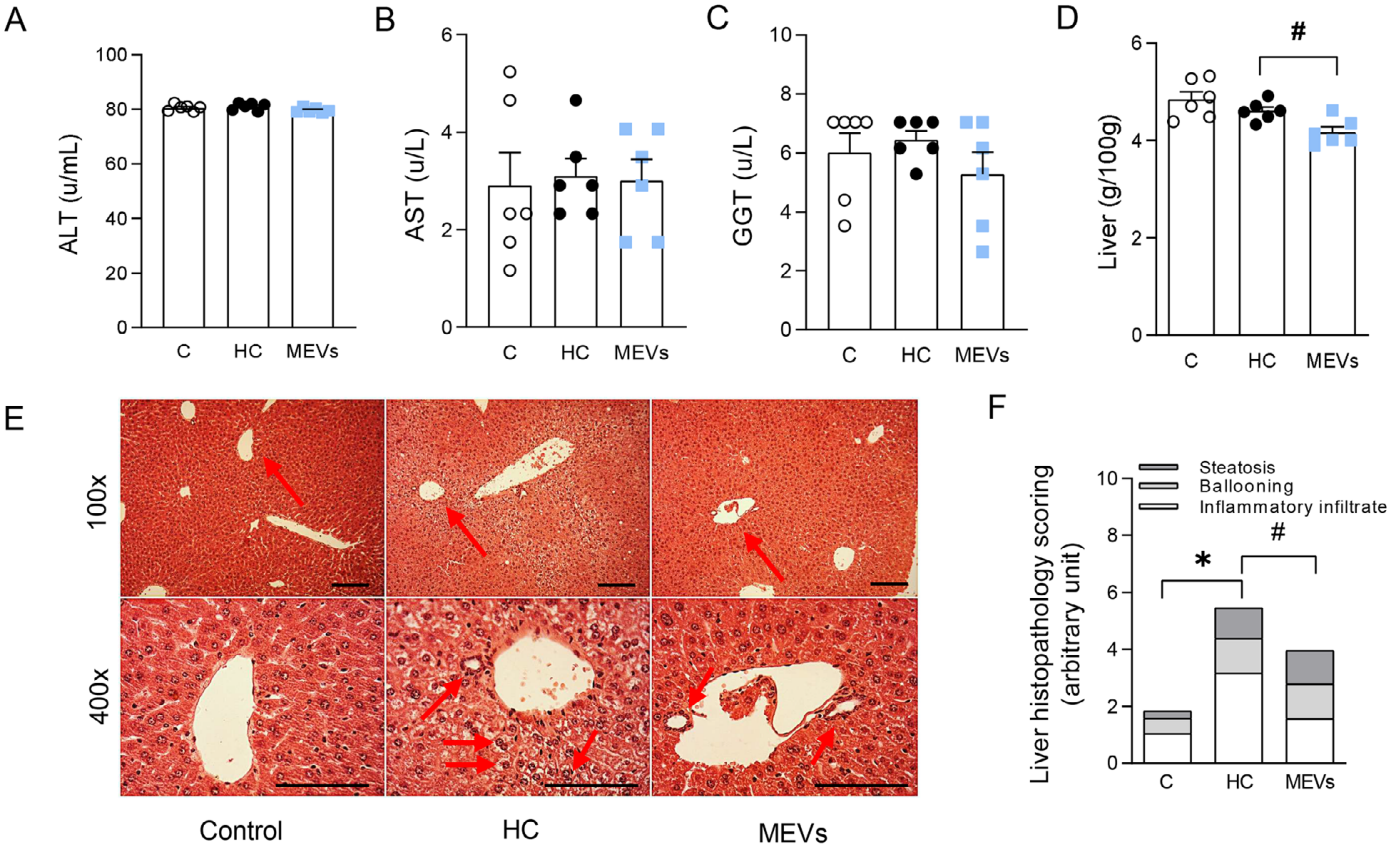
Liver histological and metabolic analysis of mice. (A) serum ALT, (B) serum aspartate aminotransferase, (C) serum gamma‐glutamyl transferase, (D) liver weight, (E) representative image of the liver histopathological score (100× and 400×), histological scale bars, 100 µm. (F) Liver histopathological score of mice fed control diet or diet rich in refined carbohydrates (HC) for 12 weeks and treated with bovine MEVs in the last 4 weeks (*n* = 6 per group). Bars represent mean values ± standard error of the mean. Statistical difference represented by *p < 0.05–HC vs. control and #*p* < 0.05–HC vs. MEVs, one‐way ANOVA, Dunnett posttest for all data. ALT, alanine aminotransferase; MEV, milk extracellular vesicle.

Mice fed the HC diet showed higher serum glucose and insulin levels (Figure 5A,B) and HOMA‐IR (Figure 5C). This impaired glucose metabolism was associated with reduced adiponectin levels (Figure 5D) and higher concentrations of triglycerides and total cholesterol in the serum than control mice (Figure 5E,F). Although mice treated with MEVs demonstrated no alterations in total cholesterol serum levels, they showed lower serum levels of glucose, insulin, triglycerides, and HOMA‐IR compared to the untreated HC group and increased adiponectin levels (Figure 5A–F). Serum ALT, AST, and GGT levels were not altered among the groups (Figure 6A–C). However, treatment with MEVs reduced the liver weight of the mice compared with the HC group (Figure 6D). Also, the liver analysis demonstrated a higher histopathological score in the liver of animals fed with the HC diet than the control. Mice treated with MEVs showed a lower tissue score, which was more related to inflammatory levels (Figure 6E,F).

## 4 Discussion

The impact of refined carbohydrates leading to bone dysfunction in both long and short bones has already been described in the literature and these effects are associated with metabolic changes [4, 6, 29]. In this study, we evaluated the effect of MEVs on bone and metabolic alterations in mice with obesity induced by an HC diet. Herein, we confirm that the HC diet increased adiposity, leading to metabolic alterations and disturbances in bone morphological and molecular parameters. Notably, treatment with MEVs counteracted these deleterious effects, improving bone and metabolic health outcomes. These data link the beneficial effects of MEVs on bone to the recovery of adipose and systemic metabolism.

In our study, mice fed the HC diet showed lower bone mass in the maxilla and femur, two bones with distinct characteristics in terms of metabolism and response to stimuli. The maxilla contains trabecular bone with a high remodeling rate, while the femur is predominantly cortical, which requires deep cellular signaling due to its larger matrix volume [30]. Bone loss in both sites, although with specific characteristics, is a common phenomenon in obesity [2, 3, 4, 5, 6], supporting the relevance of evaluating both structures in our model. Interestingly, mice receiving MEVs improved parameters in trabeculae and cortical bones. These results agree with previous studies that showed the protection of MEVs against maxillary [19] and femur bone loss after ovariectomy [21] and glucocorticoid use [20]. Furthermore, these particles have also been shown to modulate bone metabolism in moderate obesity [21]. Although alveolar bone loss was observed in mice fed the HC diet, no significant differences were found in the analysis of CEJ‐ABC or dental roots in this group compared to the control. However, MEVs reduced this area, indicating protection in the tooth support, consistent with earlier studies [19]. We show for the first time that MEVs improve bone architecture not only in the femur but also in the maxilla in a moderate obesity model, expanding the evidence of their protective potential across different bone sites.

An imbalance in the presence of bone formation and resorption cells is linked to changes in bone microarchitecture [31]. Studies have already demonstrated that the HC diet alters the profile of cells in mice's bone, which is associated with tissue loss [4, 6]. Osteoblasts and osteocytes were reduced in the maxilla of obese mice compared to controls. However, a more significant number of TRAP‐positive cells were associated with the bone loss observed in obese animals, likely contributing to the development of osteoporosis. Interestingly, MEV‐treated mice presented increased numbers of matrix‐producing cells and reduced osteoclastic activity, favoring bone formation in the model of moderate obesity. The mechanisms by which MEVs may protect against bone loss have not yet been fully elucidated. However, these nanoparticles stimulate the differentiation of osteoblasts and osteocytes [21, 23] and modulate the activity of osteoclasts [23] in vitro and in vivo [19, 21] in different models of bone loss. RUNX2 is a key regulator of osteoblast differentiation and may contribute to protection against tissue loss [32, 33]. MEVs have already been reported to stimulate its expression in vitro [23, 34]. In our study, there was an increased expression of RUNX2 in the femur of animals treated with MEVs. These data support a molecular mechanism of MEVs for the observed bone preservation effects.

We also evaluated systemic markers of bone remodeling. There were higher blood levels of RANKL and a consequent higher RANKL/OPG ratio in obese mice, indicating augmented bone resorption, consistent with previous studies [4]. In contrast, MEV treatment lowered serum RANKL and increased OPG levels, reducing RANKL/OPG ratio. These markers are secreted by cells such as osteoblasts and osteocytes and thus coordinate tissue remodeling [8, 31, 35]. Thus, this modulation likely contributes to improved bone balance through systemic regulation of remodeling pathways. In addition, we also detected elevated levels of PTX3, a key inflammatory mediator, in obese animals. PTX3 plays a role in the regulation of inflammation and has been associated with metabolic disturbances and bone loss [36, 37]. The reduction in PTX3 observed after MEV treatment suggests that these vesicles exert an anti‐inflammatory effect, which may help explain their protective role in bone metabolism. This finding reinforces the link between systemic inflammation and bone remodeling, and points to the potential of MEVs in mitigating inflammatory pathways that contribute to bone deterioration.

We examined metabolic parameters to gain a broader understanding of the bone effects. Consumption of the HC diet promotes a rapid fat accumulation in mice's adipose tissue despite no change in body weight [25, 29, 38, 39]. The increase in adipocyte area and lower expression of PPARy, a transcription factor associated with adipocyte differentiation [40], reveals a process of cellular hypertrophy in obese animals, in agreement with the increase in plasma leptin [25, 41]. After treatment with MEVs, the adipocyte size in visceral and subcutaneous tissues was reduced, and metabolic parameters, including leptin, insulin, HOMA index, glucose, and triglycerides, were improved. Insulin resistance is related to excessive hypertrophy of fatty tissue and hyperglycemia. These metabolic alterations are associated with lower BMD, inhibition of osteoblastogenesis, and activation of osteoclastogenesis due to an increase in inflammation, advanced glycation end products (AGEs), and PPARγ expression in bone marrow [42, 43, 44, 45, 46]. Few studies have reported the effect of MEVs on energy metabolism [47, 48]. The treatment with milk exosomes from humans reduced serum glucose, triglycerides and cholesterol in mice with obesity induced by a high‐fat (HF) diet [47]. Thus, these changes may be related to the increase in insulin sensitivity due to smaller adipocytes, improvement in glucose metabolism, and control of inflammatory mediators [49, 50]. To our knowledge, this is the first demonstration of the MEV impact on adipose morphology and metabolic alterations in vivo. Therefore, we hypothesize that the action of these particles may be related to fat deposition, requiring further studies to investigate mechanisms associated with lipogenic, lipolytic, or inflammatory pathways.

Liver metabolism was also evaluated as part of the metabolic assessment. Excess blood lipids contribute to changes in bone tissue and a reduction in BMD, activating inflammatory pathways and resorptive cells [51, 52, 53]. Although no differences in hepatic enzymes were found in our study, a higher histopathological damage in obese animals was observed, including inflammation and steatosis, which were attenuated by MEV treatment. In agreement, treatment with human milk exosomes in an experimental model of HF diet‐induced obesity reduced liver damage through the modulation of hepatic lipogenic and lipolytic pathways [47]. Studies that evaluated the kinetics of MEVs demonstrated that the liver is one of the first organs to receive and store these particles and can benefit from them [48, 54, 55]. The relationship between obesity and changes in the liver has already been described [38, 56, 57]. Still, the axis of liver and bone dysfunction [58], as well as the influence of MEVs on hepatic metabolism [47, 48], requires deeper investigation.

In summary, the HC diet negatively influences bone health and systemic metabolism. MEVs showed a dual protective effect in this model of HC diet‐induced obesity, attenuating bone loss and improving metabolic parameters. While it is impossible to determine whether the improvement in bone profile occurred due to metabolic changes, our data support the therapeutic potential of MEVs in modulating both skeletal and systemic pathways in obesity‐associated bone disorders.

## Conflicts of Interest

The authors declare no conflicts of interest.

## Supporting information




**Supporting file 1**: mnfr70139‐sup‐0001‐SuppMat.pdf.

## Data Availability

The data that support the findings of this study are available from the corresponding author upon reasonable request.
