# Supplementary material for: Bovine Milk Extracellular Vesicles as a Preventive Treatment for Bone Dysfunction and Metabolic Alterations in Obese Mice Fed a High‐Refined Carbohydrate Diet
Source: Mol Nutr Food Res. 2025 Jul 2;69(19):e70139. doi: 10.1002/mnfr.70139 (PMC12490194; doi:10.1002/mnfr.70139)
Supplement: Supplementary file 1 — Supporting file 1: mnfr70139‐sup‐0001‐SuppMat.pdf. [file MNFR-69-e70139-s001.pdf]

**Supplementary Table 1**. Nutritional information of the experimental diets.

| Diet composition | | |
|---|---|---|
| **Component** | **Chow diet** | **High refined-carbohydrate diet** |
| Carbohydrates (%) | 65.8 | 72.2 |
| Sucrose (%) | - | 30.0 |
| Fats (%) | 3.1 | 5.8 |
| Proteins (%) | 31.1 | 20.0 |
| Energy Density (kcal/g) | 4.0 | 4.4 |

**Supplementary Table 2.** Real-time PCR primer sequence.

| Gene | Forward | Reverse |
|---|---|---|
| **RUNX2** | GGCCGGGAATGATGAGAACTA | CAGATCGTTGAACCTGGCTACTT |
| **PPARy** | ATCTCCGCCAACAGCTTCT | TAACTGCCGGATCCACAAA |
| **GAPDH** | ACGGCCGCATCTTCTTGTGCA | CGCCCAAATCCGTTCACACCGA |

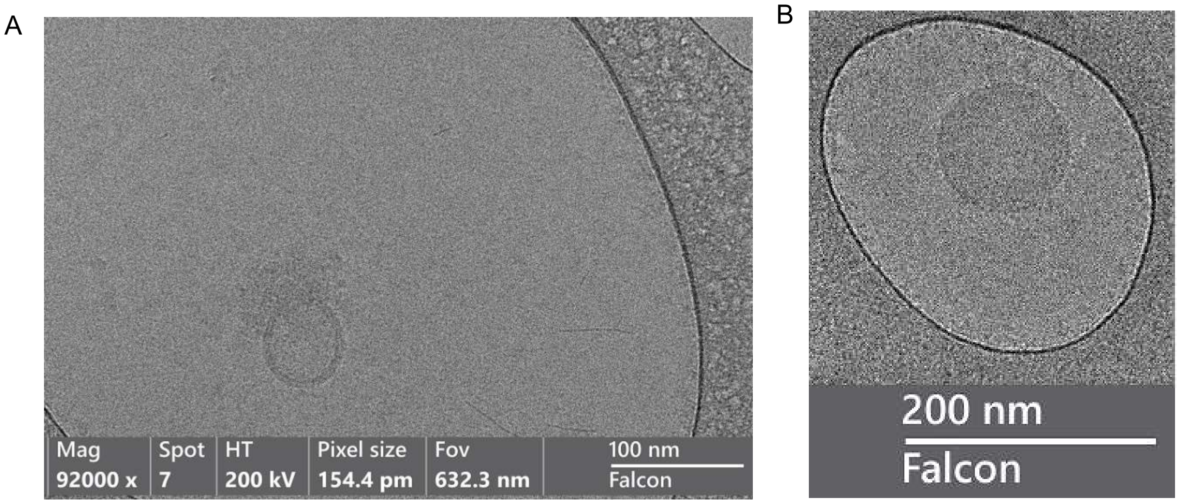


**Supplementary Figure 1 - Images of bovine milk extracellular vesicles by cryo-electron microscopy (cryo-EM).** (A, B) Electrolucent bovine milk extracellular vesicle with a rounded shape and a well-defined lipid bilayer.

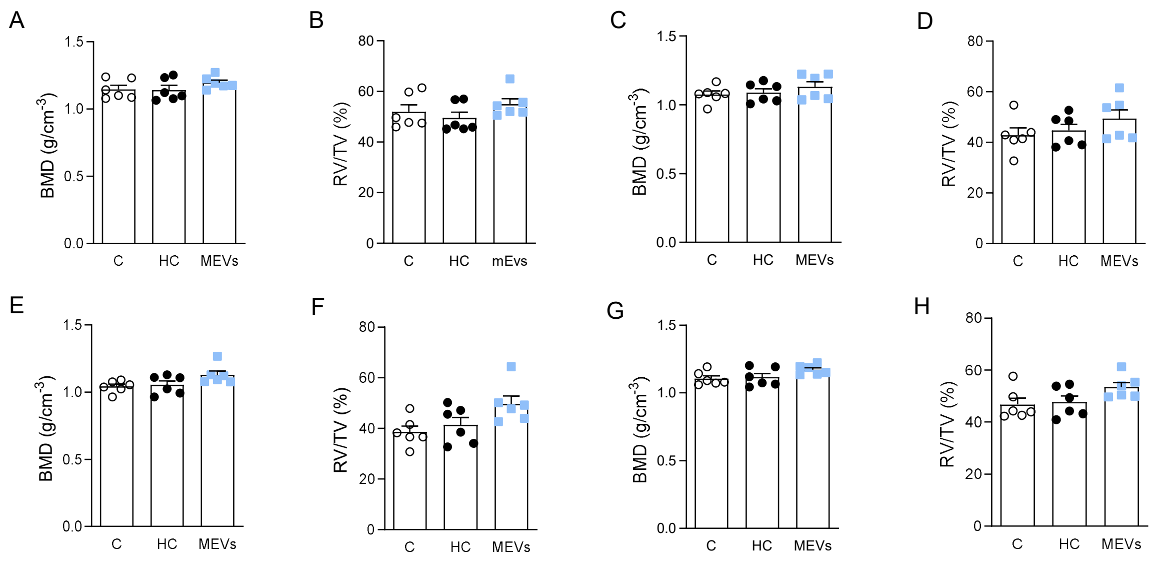


**Supplementary Figure 2 - Analysis of the maxillary roots of mice fed with HC diet and treated with MEVs by computed microtomography.** (A) Bone mineral density (BMD) of the mesiobuccal root, (B) Percent bone volume/tissue volume (RV/TV) of the mesiobuccal root, (C) Bone mineral density (BMD) of the palatal root, (D) Percent bone volume/tissue volume (RV/TV) of the palatal root, (E) Bone mineral density (BMD) of the distobuccal root, (F) Percent bone volume/tissue volume (RV/TV) of the distobuccal root, (G) Bone mineral density (BMD) of all maxillary roots, (H) Percent bone volume/tissue volume (RV/TV) of all maxillary roots of mice fed the control diet or diet rich in refined carbohydrates (HC) for 12 weeks and treated with extracellular milk vesicles (MEVs) in the last 4 weeks. Bars represent mean values ± standard error of the mean (n=6). One-way ANOVA, Dunnett posttest, $p>0.05$.
